# We Need a Combination of Approaches to Evaluate Health System Resilience: Comment on "Re-evaluating Our Knowledge of Health System Resilience During COVID-19: Lessons From the First Two Years of the Pandemic"

**DOI:** 10.34172/ijhpm.8564

**Published:** 2024-07-20

**Authors:** Julia Zimmermann, Marina Karanikolos, Jonathan Cylus, Martin McKee

**Affiliations:** ^1^European Observatory on Health Systems and Policies, London School of Economics and Political Science, London, UK.; ^2^European Observatory on Health Systems and Policies, London School of Hygiene and Tropical Medicine, London, UK.; ^3^London School of Hygiene and Tropical Medicine, London, UK.

**Keywords:** Resilience, Health System, Health System Resilience, Health System Performance, Health Security, Preparedness Planning

## Abstract

Health system resilience has become a desirable health system attribute in the current permacrisis environment. The article by Saulnier and colleagues reviews the literature on health system resilience and refines the concept, pinpointing dimensions of resilience governance that have not reached consensus, or that are missing from the literature. In this commentary we complement the findings by discussing different conceptual frameworks for understanding resilience and introducing resilience testing, a method to assess health system resilience using a hypothetical shock scenario. Resilience testing is a mixed-methods approach that combines a review of existing data with a structured workshop, where health system experts collaboratively assess the resilience of their health system. The new method is proposed as a tool for policy-making, as the results can identify attributes of the current health system that may hinder or boost a resilient response to the next crisis.

## Introduction

 In the accompanying paper, Saulnier and colleagues^1^ take a fresh look at the concept of health system resilience, synthesizing what we have learned from the pandemic. In an increasingly uncertain world beset by the effects of climate change and its many consequences, where post-war mechanisms for global governance and rules-based order are under threat,^2^ the need for health systems to be able to withstand shocks is self-evident.^3^ Applying the idea of resilience to health and health systems is not new and has been discussed by those responsible for emergency planning for several decades, especially after a series on of natural and man-made disasters in the 2000s.^4^ Resilience has, however, gained much greater prominence in the aftermath of the COVID-19 pandemic when it became clear that some institutions and communities were much more resilient when facing the shock than others.^5^

 After reviewing the literature on health system responses to the pandemic, Saulnier and colleagues^1^ mapped the issues they identified onto a governance framework with four dimensions, (using) knowledge, (coping with) uncertainty, interdependence, and legitimacy. Collectively these dimensions may enable any health system to absorb and adapt to a shock and to transform in response to it.^6^ Saulnier et al then explored the extent to which the range of issues identified were (or were not) described by the resilience literature, identifying gaps. For example, the literature on teamwork and values was found to have gaps and the ability of a health system to absorb and adapt to shocks was better described than the ability to transform.

 Saulnier and colleagues’ paper makes an important contribution to what is now a substantial body of writing from a variety of disciplines on health system resilience, which has historically considered many types of shocks, from terrorist attacks to political crises and earthquakes to volcanic eruptions. However, reflecting on the diversity of shocks, multiple disciplines and fields of research that have studied them and the silos in which these groups often operate,^7^ the health system resilience literature is complicated by many different definitions of health system resilience, none of which are universally accepted.^8^ Consistent with the findings of Saulnier and colleagues,^1^ the health system resilience literature tends to focus on the capacity of health systems to absorb a shock and adapt to it rather than to transform.^9^

 While Saulnier et al have made an important contribution to unifying the thinking underpinning different definitions of health systems resilience, they conclude by writing that “it would be worthwhile to conduct further analyses using other frameworks, to ascertain areas of overlap between concepts and to generate a more comprehensive assessment of where the concept of health system resilience currently stands.” In this commentary we have taken up their invitation.

 This commentary complements Saulnier and colleagues’ article by reflecting on different conceptual frameworks that can help understand health system resilience and exploring how health system resilience can be useful, not only for retrospective but also prospective analysis. We draw on *Strengthening Health Systems: A Practical Handbook for Resilience Testing*, which describes a methodology for resilience testing that was developed jointly by the European Observatory on Health Systems and Policies and the Organisation for Economic Co-operation and Development, and funded by the European Commission.^10^ This work has been informed by a review of existing research and practice, and piloted by national and regional governments in Europe.

 The resilience testing handbook sets out a five-step process that starts with a period of preparation, where the details of the resilience test are agreed, and existing data is reviewed.^10^ This is followed by a resilience test day, which is a structured workshop that brings together relevant stakeholders from the health system and other relevant sectors, who systematically assess resilience of the health system in the context of a hypothetical shock scenario. The intended outcome of the resilience test is the identification of actionable system-level weaknesses that may undermine the health system’s response to the specific shock, but also have broader implications on health system functioning. The knowledge gained through a resilience test can be used to design remedial policy action, to improve the resilience of the health system going forward.

 In the paragraphs that follow, we describe the lessons that we have learned while developing the resilience testing process.

## Resilience as a Dynamic Element of Health System Performance

 Resilience can be considered as an attribute of the health system that can be captured through a range of health system performance measures. Consequently, our starting point is a performance framework, the Global Health System Performance Assessment (HSPA) framework, developed by the European Observatory on Health Systems and Policies and the World Health Organization (WHO).^11^ Compared with the approach taken by Saulnier et al, with its emphasis on governance, this allows us to look at other health system functions, such as financing, resource generation and service delivery. The Global HSPA framework draws links between the different health system inputs, the health system outputs and outcomes, and facilitates understanding of how different functions impact on each other. It was designed to be both comprehensive and flexible, so that it can be applied easily to different health system contexts. It also ensures that resilience assessment covers structural and functional issues, such as the availability and quality of human and physical resources and adequacy of financing. From this perspective, if a health system is to be described as high performing it must be able to demonstrate that it is resilient in the face of shocks, a state that it can only achieve if its different elements are themselves performing well.

 Health system resilience is dynamic rather than static. A resilient health system is one that can maintain its performance throughout the shock, which may have effects that endure for a substantial period, and continue into the recovery period.^12^ As Saulnier et al note, most of the papers they reviewed focused on the shock itself, with only limited consideration of the pre-existing state of the health system or recovery from the effects of the shock. Similarly, HSPA frameworks are not designed to capture changes over time and therefore may miss important considerations. To mitigate this, the resilience testing methodology described in *Strengthening Health Systems: A Practical Handbook for Resilience Testing* combines the global HSPA framework with the shock cycle framework. The shock cycle framework contains four stages that describe how health systems experience a shock over time.^12^ These are: (*i*) preparedness; (*ii*) shock onset and alert; (*iii*) shock impact and management (that includes capacity to absorb, adapt and transform), and (*iv*) recovery and learning. Combining this framework with the global HSPA framework allows for a comprehensive evaluation of all aspects of health system functioning at each stage of the shock cycle.

 The question then is whether this approach works? We have undertaken a series of exercises in different European countries and contexts and found that this combination of the shock cycle and the global HSPA framework provides a robust conceptual basis for analyzing resilience. The combination of frameworks provides structure and facilitates a systematic approach to analyzing the health system, while the resilience testing method maintains the flexibility to explore the impact of contextual factors that lie outside of the scope of the health system. For example, a resilience test that was recently conducted in Finland included discussions on values, ethical considerations and the political determinants of the health system response to a shock, issues that Saulnier and colleagues’ review flagged as relatively unexplored.^13^

## Using Health System Resilience Testing to Prepare for Future Shocks

 As Saulnier and colleagues’ note, there is consensus that the lessons that can be learnt from one type of shock may be transferrable to others. Resilience testing exploits this potential to learn to improve health system resilience. Instead of an actual shock, resilience testing uses a hypothetical shock scenario. By bringing together relevant stakeholders with expertise in the day-to-day functioning of the health system, it is possible to identify potential areas of weakness, and ways to address them. Similar to retrospective resilience reviews, the resilience testing process is designed to identify desirable and undesirable attributes of existing systems and characteristics of processes that may hinder or enhance a resilient response. This creates a learning process specific to the national context that can be used to improve the resilience of the health system for an actual shock.

 The importance of taking a broad perspective became very clear in the pandemic, with many responses paying little attention to the indirect effects of the shock and its response.^14^ Resilience testing complements existing, often narrower, approaches to health security requirements, such as those used to monitor implementation of the International Health Regulations, and contributes to implementation of the EU regulation (2022/2371) on serious cross-border health threats, which envisages stress testing and simulation exercises to help health systems prepare for the next shock.^10^

 The evidence used to support a resilience test includes information on contemporary health system performance and on responses to previous shocks. Resilience testing requires the collection, synthesis, and evaluation of data, both routine and bespoke, compiled in the run-up to the resilience test day. The data analysis can be informed by a series of resilience indicators that have been compiled by the WHO^15^ or by example indicators described by the handbook for resilience testing. Crucially, the conclusions of a resilience test may include the need for new and better data systems going forward.

 The main limitation of health system resilience testing is that is uses a formative rather than summative approach due to the hypothetical nature of the shock. This approach has limited ability to detect unanticipated outcomes (“unknown unknowns”). Further, pragmatic choices must be made when prioritizing the parts of the health system that are most relevant to the shock scenario and the wider context. These choices are prone to uncertainty and bias and resemble the choices made by researchers who conduct a retrospective analysis of resilience; as Saulnier and colleagues’ review shows,^1^ some dimensions of resilience are largely absent from the literature. In a resilience test, these choices may be dictated by a number of factors, including the time and resources available, the availability of information, individual prioritization or the level of controversy expected in the discussions. It therefore is important that those organizing resilience tests are highly knowledgeable about the health system and have adequate expertise, are seen as politically neutral, and are able to challenge the status quo. It is also important that stakeholders invited to participate in the resilience test represent a wide range of relevant health system functions, are able to criticize their own work constructively, but can also identify interdependencies and system-wide bottlenecks.

## Conclusion

 Health system resilience is now recognized as an important characteristic of a high performing health system, albeit one that is still being debated as the different definitions, conceptual frameworks and approaches to operationalizing resilience are resolved and aligned. Resilience testing adds yet another approach to this evolving body of work. Reviews such as that conducted by Saulnier and al. contribute importantly to this process of consolidating and integrating the different approaches to health system resilience, identifying where there is consensus and where further work is needed.

## Ethical issues

 Not applicable.

## Competing interests

 Authors declare that they have no competing interests.
